# Heavy Metals and Probabilistic Risk Assessment via *Pheretima* (a Traditional Chinese Medicine) Consumption in China

**DOI:** 10.3389/fphar.2021.803592

**Published:** 2022-01-05

**Authors:** Xiaohui Xu, Limin Li, Heng Zhou, Qing Hu, Lingling Wang, Qiang Cai, Yin Zhu, Shen Ji

**Affiliations:** ^1^ Yangtze Delta Region Institute of Tsinghua University, Zhejiang, China; ^2^ Shanghai Institute for Food and Drug Control, NMPA Key Laboratory for Quality Control of Traditional Chinese Medicine, Shanghai, China; ^3^ Shandong Academy of Medical Sciences, Shandong, China

**Keywords:** pheretima, traditional Chinese medicine, heavy metals, probabilistic risk assessment, Monte Carlo simulation

## Abstract

Earthworms are known to accumulate inorganic contaminants from the soil; they are also used as a traditional Chinese medicine (TCM) called *Pheretima*, which might cause safety problems with long-term exposure. Here, this study was conducted to determine and analyze the level of heavy metal contamination such as arsenic (As), cadmium (Cd), chromium (Cr), copper (Cu), mercury (Hg), manganese (Mn), nickel (Ni), and lead (Pb) in *Pheretima* and then explore the probabilistic health risks caused by 8 heavy metals in 98 batches of *Pheretima* using Monte Carlo simulation. A risk assessment strategy was conducted to assess heavy metal–associated health risk of *Pheretima* based on consumption data. For random consumption sampling, the results found that the non-carcinogenic risk of As is higher than the acceptable level, and the carcinogenic risk levels of As and Cr exceeded the acceptable risk recommended by the USEPA. Cr and As were regarded as the priority metals for risk control in the present study. Finally, it was recommended that the dosing frequency should be less than 24 d/y. In general, this study conducted a probabilistic risk assessment of heavy metals in *Pheretima*, which would be of significance for policy makers to take effective strategies to improve the quality and safety of *Pheretima*.

## 1 Introduction

Heavy metals have attracted much attention because of their undesirable effects and toxicological manifestations (Si et al., 2015; Islam et al., 2020). A wide range of anthropogenic activities could introduce heavy metals into the environment, including coal combustion, ore processing, mining, metal-enriched pesticide application, chemical fertilizer use, leather tanning, and electroplating (Hanfi et al., 2019; Mishra et al., 2021). The uncontrolled release of heavy metals into the soil environment results in harmful effects on human health through bioaccumulation in the food chain (Adimalla, 2020; Hou et al., 2020). Previous studies have shown that heavy metals can enter the human body via food and water, leading to severe health hazards in terms of developmental abnormalities, reproductive abnormalities, and reduction in life span (Ali et al., 2019; Paithankar et al., 2021).

Traditional Chinese medicines (TCMs) have been an integral part of Chinese culture and the primary medical treatment for a large portion of the population for thousands of years (Coghlan et al., 2012). However, TCMs are not risk-free because of undeclared or misidentified TCMs’ ingredients including allergenic substances (Ernst, 2000), plant toxins (Still, 2003), heavy metal (Ernst, 2002; Harris et al., 2011), and pharmaceutically active compounds of undetermined concentration (Sakurai, 2011; Coghlan et al., 2012). Numerous case reports and case series related to heavy metal poisoning attributable to the use of traditional Chinese medicine (TCM) have been published (Ernst and Coon, 2001). The main reasons of heavy metal pollution in TCMs are the accumulation of heavy metals in the environment and inadvertent pollution during the production process (Zhang et al., 2012). Luo et al. (2020) detected 1773 samples of TCM around the world, and the concentrations of Pb, Cd, As, Hg, and Cu exceeded the standard in the Chinese Pharmacopoeia 2020 (ChP). Some heavy metals (Cr, Ni, Cu, and Mn) are essential micronutrients for plants and animals. However, they become toxic at higher concentrations (Filipiak-Szok et al., 2015). For instance, the maximum concentration of Mn in a *Coix* seed from a Chinese local medicine store was 32.30 mg/kg, which exceeded the limit (Yang et al., 2020). In 2014, some studies have pointed out the maximum content of Cr in *Scutellariae radix* from Heilongjiang Province was 84.76 mg/kg (Chen et al., 2021). Besides, heavy metal residues will persist in the TCM even after boiling, causing heavy metal toxicity (Ernst, 2002). However, few studies have been conducted on a wide range of TCMs, the quality of which is closely related to the patient’s health as patients may be more vulnerable to the intake of heavy metals. Therefore, it is crucial to determine the level of heavy metals in TCMs to evaluate the health risk of TCMs.

Earthworms are important deposit feeders in terrestrial ecosystems and can accumulate contaminants from the soil (Huang et al., 2017). They are thus commonly considered important indicator organisms for risk assessment (Nyholm et al., 2010; Xie et al., 2013). The dried body of an earthworm is a type of TCM, called *Pheretima* (ChP), which is said to possess multiple bioactivities, such as antipyretics, antiasthmatics, anticoagulants, and diuretics (Sun et al., 2019; Wu et al., 2019). There are limited publications focusing on heavy metal contaminated in TCMs and associated with risk assessments on websites. Long-term exposure of heavy metals via the consumption of *Pheretima* would inevitably cause chronic health risks. However, scarce data on heavy metal contamination in *Pheretima* are available, and limited research has investigated the probabilistic health risks assessment of *Pheretima*.

Previous studies have used a deterministic method to assess the health risk of heavy metals in TCM but ignored the uncertainty of the concentration of heavy metals and various exposure parameters. For instance, Zuo et al. (2019) evaluated the risk of heavy metals in traditional animal medicines using a deterministic health risk assessment method and then found that the cancer risk of As exceeded the acceptable lifetime risk in *Pheretima*. However, some limitations of the current studies need to be acknowledged. For one thing, the consumption data used in those studies were not the specific TCM but the total of TCM. For another thing, the existing reports only evaluate the health risk levels based on a single value (median, mean, or P95) and ignore the uncertainty of hazardous concentrations (Zuo et al., 2020), which may affect the accuracy of final results. Therefore, some measures were taken in the current study to address these limitations. The probabilistic risk assessment method can provide more information than the deterministic health risk assessment method. Therefore, in this study, a probabilistic approach using Monte Carlo simulation was selected to investigate the health risk of *Pheretima*.

In general, this study intended to explore multiple heavy metal concentrations in *Pheretima*-associated human risk assessment in the Chinese population. The objective of this research were 1) to investigate As, Cd, Cr, Cu, Hg, Mn, Ni, and Pb concentrations in *Pheretima* across China using inductively coupled plasma mass spectrometry (ICP-MS), 2) to conduct the probabilistic risk assessment of both non-carcinogenic and carcinogenic risks for exposure to heavy metals via the consumption of *Pheretima*, and 3) to provide recommended exposure frequency for *Pheretima* consumption based on the maximum recommended the daily intake and the maximum metal concentration limit in Chinese Pharmacopoeia 2020 (ChP).

## 2 Materials and Methods

### 2.1 Consumption Data and Samples Collection

To ensure a realistic assessment of the health risks associated with *Pheretima* intake, a consumption data analysis was conducted in Shanghai. The consumption data of Chinese medicine were from Shanghai hospitals and pharmacies in 2019. The people involved in these data were older than 18 years, and their names have been anonymized. After data cleaning and analysis, the result showed that a total of 29,216 individuals purchased *Pheretima*.

To provide insights into heavy metal occurrence in *Pheretima*, all the 98 samples of *Pheretima* were collected from different medicinal material markets and pharmacies in China (Anhui, Sichuan, Heibei, Guangxi, Shandong, and Shanghai). All the samples were processed using morphological and histological methods according to the Chinese Pharmacopoeia 2020 (ChP) standards, marked with their origins and categories. Voucher specimens were deposited in the Shanghai Institute for Food and Drug Control. All the samples were sterilized with 75% ethanol and then kept dry in moisture-proof plastic bags at 4°C until analysis before grinding to a fine powder.

### 2.2 Samples Digestion and Analysis

According to the Chinese Pharmacopoeia 2020 (ChP), each sample was powdered and homogenized using a ball-milling instrument at 4°C. Microwave-assisted dissolution of *Pheretima* using a closed vessel system was performed on 0.2 g of the earthworm homogenate (dry weight). Then 4 ml of nitric acid and 1 ml of hydrochloric acid were added to each vessel before allowing it to stand for 30 min, which is followed by microwaving. Following an initial heating procedure (ramping up to 75°C over 5 min, then holding for 1 min; ramping up to 100°C over 3 min, then holding for 3 min; ramping up to 150°C over 7 min, then holding for 3 min; ramping up to 170°C over 5 min, then holding for 3 min; and ramping up to 190°C over 5 min, then holding for 10 min), the samples were cooled below 60°C. Then acid in the tubes was dispelled for 40 min and made up to 50 ml with Milli-Q water. Finally, all samples were measured using the Agilent 7700X ICP-MS (Agilent 7700X, Agilent Technologies Co., United States) to measure the contents of As, Pb, Ni, Cr, Cu, Cd, Mn, and Hg (see Supplementary Table S1 for operating parameters of Agilent 7700 ICP -MS).

### 2.3 Health Risk Assessment of Heavy Metal

#### 2.3.1 Estimated Daily Intake

When using a single-point value to estimate health risk of exposure to pollutants such as heavy metals, the probability of interference and error, and eventually the uncertainty of the result, is achieved. Therefore, in the present research, a probabilistic analysis with Monte Carlo simulation was employed to reduce the uncertainty of estimation (Fallahzadeh et al., 2018).

Health risk assessment, including non-carcinogenic and carcinogenic risks, is widely used to quantitatively estimate the probability and the probable degree of pollutants on human health (Man et al., 2010; Pena-Fernandez et al., 2014). The expression used to evaluate the exposure dose can be calculated using Eq. 1:

$$\begin{array}{c} EDI=\frac{C_{i}\times AC\times E_{D}}{BW\times T_{A}\times 1000}\times T \end{array},$$ (1)

where *EDI* represents the estimated intake dose per kilogram of body weight for a long time (mg/kg bw day^−1^); *C*_*i*_ (mg kg^−1^) is the concentration of the heavy metal i in rice; *AC* (g year^−1^) is the annual consumption of *Pheretima*; *E*_*D*_ (year) is the exposure duration; and *BW* is the average weight of the exposed population (63 kg), kg. According to the National Health and Family Planning Commission of the People’s Republic of China, the average weight of Chinese adult is 63 kg in 2015; *T_A_* is the average exposure time or period of exposure (days), *E*_*D*_ × 365 days year^−1^, and 70 × 365 days year^−1^ for non-carcinogenic and carcinogenic risks, respectively (EPA, 2011). *T* represents the transfer ratio of heavy metals from *Pheretima* materials to decoction or preparations (%). TCM is used to prepare decoctions, and this process can contribute to the dissipation of heavy metals, resulting in lower risk (Harris et al., 2011). It is assumed that the transfer ratio is 10% in this study (Zuo et al., 2020).

#### 2.3.2 Non-Carcinogenic Risks and Carcinogenic Risks

The health risk of heavy metals can be calculated based on *EDI*. The hazard quotient (*HQ*) and *CRi* are taken as measures of non-carcinogenic and carcinogenic risk assessment, respectively (Li et al., 2021). *HQ* can be expressed as the ratio of daily exposure dose to the reference dose. *CRi* represents the carcinogenic risk of an individual metal. When the population is exposed to two or more pollutants, the hazard index (*HI*) is adopted to evaluate the non-carcinogenic health risk of these multiple chemical pollutants. Similarly, the *CRi* can be added to generate a *CR*_*T*_ to estimate the integrated carcinogenic risk of mixing heavy metals. The health risks were calculated by the following equations (Eqs 2–5) (Kukusamude et al., 2021):

$$\begin{array}{c} HQ=\frac{EDI}{RfD} \end{array},$$ (2)

$$\begin{array}{c} HI=\sum HQ \end{array},$$ (3)

$$\begin{array}{c} CR_{i}=EDI\times CSF \end{array},\;$$ (4)

$$\begin{array}{c} CR_{T}=\sum_{i}^{n}Risk_{i} \end{array}.$$ (5)

If the *HQ* or *HI* exceeds 1, there would be a certain degree of adverse effects on human health; otherwise, non-carcinogenic risk for human being is acceptable. The total non-carcinogenic *HI* was calculated in accordance with Eq. 3. *RfD* is the reference dose of non-carcinogenic metals in *Pheretima*, mg/(kg d). *RfDs* for Cr, Mn, Co, Ni, Cu, Zn, Cd, As, Pb, Al, and Hg are 1.5, 0.14, 0.0003, 0.02, 0.04, 0.3, 0.001, 0.0003, 0.0085, 0.0004, and 0.0003 mg/(kg d), respectively (Song et al., 2015; Lim et al., 2018). As shown in Eq. 4, *CR_i_* is the hazard quotient of trace metal through ingestion. *CSF* is the oral carcinogenic slope factor of carcinogenic metals (kg d)/mg. *CSFs* for Cr, Cd, Pb, and As are 0.5, 0.38, 0.0085, and 1.5 (kg d)/mg, respectively (Lim et al., 2018; Zhang et al., 2019). In general, the acceptable *CRi* standard ranges from 10^−6^ to 10^−4^ (USEPA, 2005). Therefore, if *CR*_*i*_ and *CR*_*T*_ values are higher than 10^−4^, the carcinogenic risk over a lifetime is considered unacceptable.

### 2.4 Quality Assurance and Quality Control

Quality assurance and quality control were strictly carried out with parallel samples, method blanks, and reference material recovery tests (Zuo et al., 2020). In order to remove the background values of trace metals in all the reagents and containers, all the glassware and plastic vessels used in the present study were previously soaked in 10% (v/v) HNO_3_ solution for more than 24 h and rinsed three times with Milli-Q water before using. During the experiment, the contact of all the samples with metal-based materials was avoided. The accuracy of the analytical method was validated using spike recovery methods (Xu et al., 2012). The results were quantified with an empirical calibration curve using a multi-element calibration standard material (GNM-M27195-2013) obtained from Chinese National Standard Reference Center. The recoveries for all heavy metals in Pheretima ranged from 80.1 to 113.0%. A standard curve was evaluated for each trace element, and the results of the correlation coefficient (R_2_) were greater than 0.99. Method blanks were performed in each batch of samples, and the process was carried throughout whole sample preparation and analytical procedures (Roda et al., 2019). These blanks were useful to ensure little or no background metal interference during digestion and determination. The relative standard deviation (RSD) of replicate analysis of samples was within 15%. Detailed parameters could be found in supplementary materials (Supplementary Table S2).

### 2.5 Statistical Analysis

Concentrations lower than the LODs were reported as not detected (ND) and replaced by 0 for data analysis, while concentrations lower than the LOQs but higher than LODs were calculated as 1/2 the LOQs (Chen et al., 2014). Monte Carlo simulation was performed for 10,000 iterations to quantify the uncertainty of the measured contaminant concentrations and annual consumption in health risk evaluation using the sample function in R. Point value data were transformed into statistical random variables. The probability distribution of the desired parameters was evaluated. Data management and analysis were performed using Program R (version 4.0.3) and plotted using the package ggplot2 (https://ggplot2.tidyverse.org/). The “fitDistr” function in propagate R package was applied for concentrations of selected trace elements in *Pheretima* (Delignette-Muller and Dutang, 2015).

## 3 Results

### 3.1 Consumption Characteristics of *Pheretima*

The availability of detailed and high-quality consumption data collected at an individual level is essential for assessing the exposure to potential risks (Nelis et al., 2018). After the analysis of consumption records in Shanghai pharmaceutical factories and hospitals, a total of 29,216 people consumed *Pheretima* in 2019. As is shown in Table 1, the mean and 95th percentile of exposure duration of *Pheretima* intake were 30.4 and 98 days per year, respectively. The mean and 95th percentile of annual consumption were 318.9 and 1,050 g/day, respectively. In the probabilistic assessment of this study, a value of annual consumption data will be randomly selected from the dataset of *Pheretima* consumption in order to complete 10,000 iterations.

**TABLE 1:** Consumption characteristics of *Pheretima*.

Parameter	Annual consumption (g/y)	Exposure frequency (d)	Daily intake rate (g/d)
P5	60.0	7.0	6.0
P50	168.0	14.0	9.0
P95	1,050.0	98.0	18.0
Mean	318.9	30.4	10.3

### 3.2 Concentrations of Heavy Metals in *Pheretima* in Chinese Markets

**FIGURE 1:**
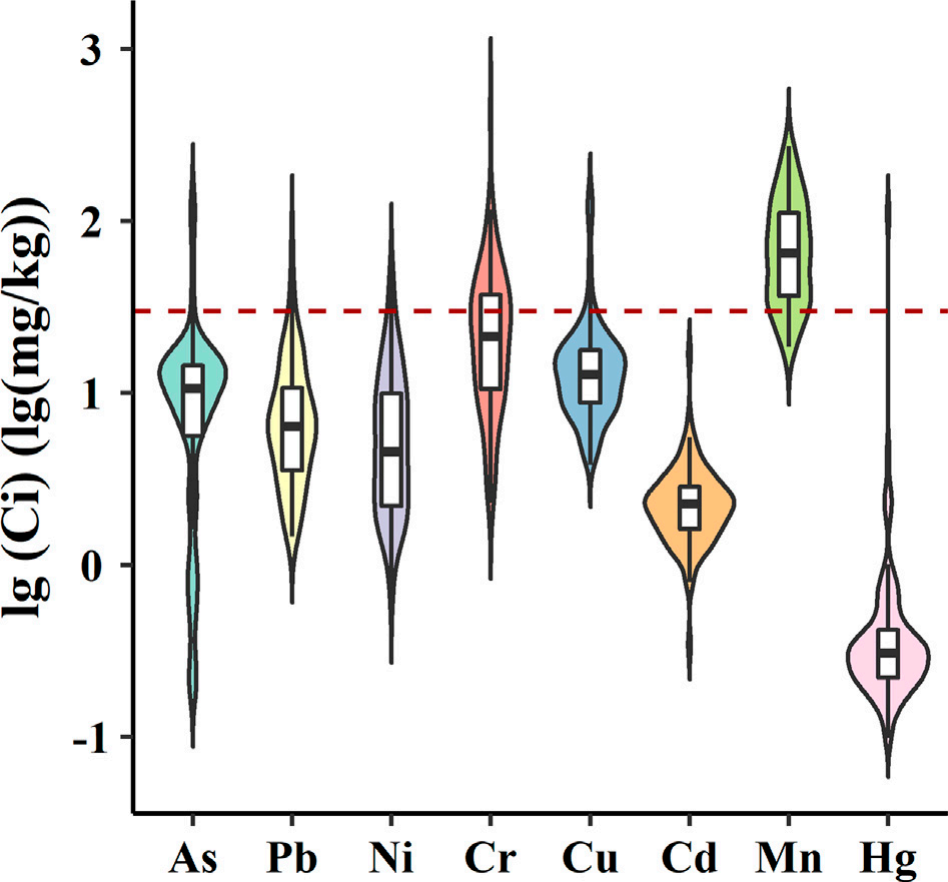
Concentration of eight heavy metals in *Pheretima*. The dashed line represents the limits set in the Chinese Pharmacopoeia 2020 (ChP).

The concentrations of eight heavy metals in *Pheretima* are displayed in Figure 1. All heavy metals were detected in every sample. Mean total contents of As, Cd, Cr, Cu, Hg, Mn, Ni, and Pb were 13.51, 2.52, 31.85, 17.23, 1.58, 83.45, 8.05, and 9.33 mg/kg, respectively. They were found in the following decreasing order: Mn > Cr > Cu > As > Pb > Ni > Cd > Hg. Thus, Mn, Cr, and Cu were the three major heavy metals. According to the Chinese Pharmacopoeia 2020 (ChP), the maximum residual limit of heavy metals in *Pheretima* is 30 mg/kg. The exceedance ratios of As, Cd, Cr, Cu, Hg, Mn, Ni, and Pb in *Pheretima* were 5.10, 0, 35.71, 6.12, 1.02, 82.65, 3.06, and 3.06%, respectively. In terms of the exceedance ratios, the *Pheretima* samples were more heavily contaminated with Mn and As than with the other metals. Based on the results of the fitdistr package in R language, the concentration of As was fitted with the Weibull distribution, the concentration of Mn was fitted with the inverse Gaussian distribution, and the concentrations of the other six metals were fitted using the log-normal distribution. The parameters of the fitted distribution of metal concentrations are also shown in Supplementary Table S3. Earthworms, the raw material of *Pheretima*, could accumulate various organic and inorganic contaminants present in the soil (Jager et al., 2003). Thus, further attention should be paid to heavy metals in *Pheretima*.

### 3.3 Health Risk Assessment of Exposure to Heavy Metals

The non-carcinogenic risks of eight heavy metals and cancer risks of four heavy metals were calculated by using Eqs 1, 2, and 4. To complete 10,000 iterations in the probabilistic assessment, the value of annual consumption data was randomly selected from the dataset of *Pheretima* consumption. With the probability distribution of *Ci*, Monte Carlo simulation was applied to determine the uncertainty in the risk assessments.

#### 3.3.1 Non-Carcinogenic Risk Assessment of Heavy Metals in *Pheretima*

**FIGURE 2:**
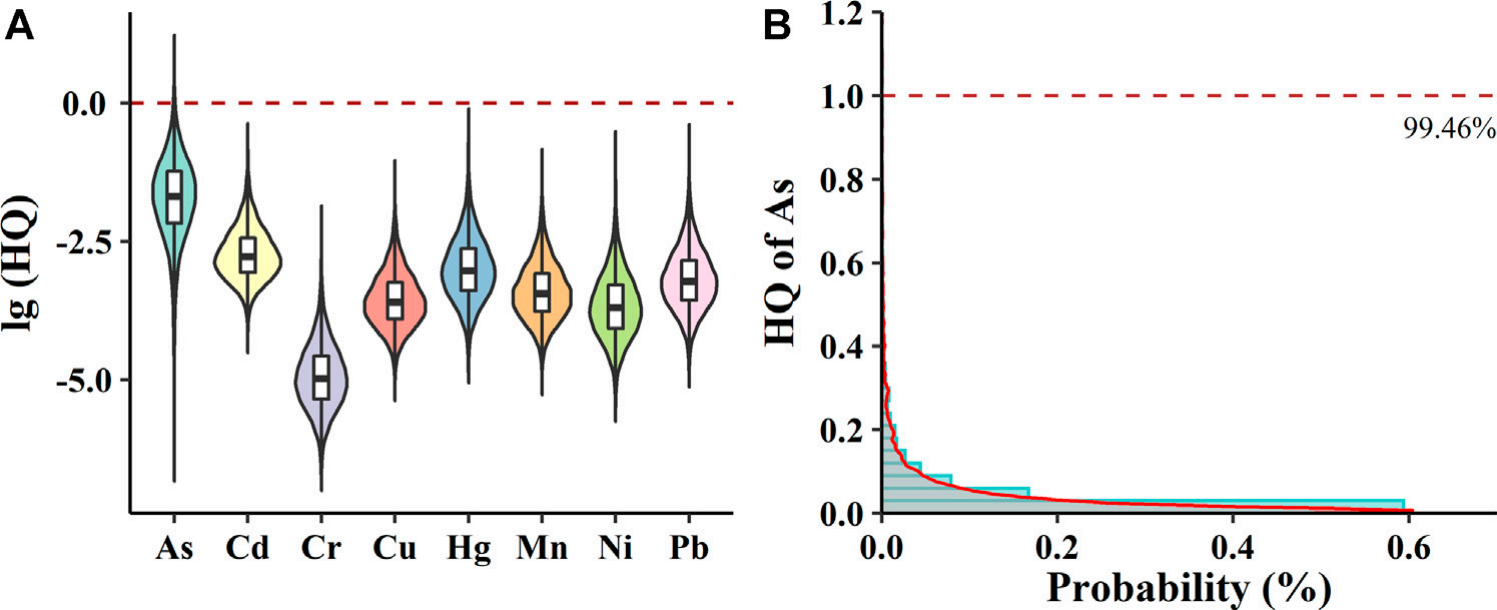
Estimated non-carcinogenic risks (HQ) of selected heavy metals in *Pheretima*. **(A)** Violin plot showing log10 (HQ) values of eight heavy metals in *Pheretima*. **(B)** The probability distribution of potential health risk of As. The red dashed line represents the acceptable limit of HQ.

Based on a Monte Carlo simulation using 10,000 iterations, the maximum values of non-carcinogenic risks for As, Cd, Cr, Cu, Hg, Mn, Ni, and Pb were 8.50, 1.21 × 10^−1^, 2.15 × 10^−3^, 2.00 × 10^−2^, 1.62 × 10^−1^, 3.64 × 10^−2^, 1.12 × 10^−1^, and 5.58 × 10^−2^, respectively. Figure 2A displays the violin and boxplot of non-carcinogenic risk levels of the selected heavy metals based on probabilistic health risk assessments in *Pheretima*. All HQ values of Cd, Cr, Cu, Hg, Mn, Ni, and Pb for 98 batches of *Pheretima* were lower than 1, which is safe for humans. Data regarding the risks associated with As exposure were of great concern in the present study. As exists in different organic and inorganic forms; however, different As speciation possesses different levels of toxicity. Based on the need to protect most consumers, it was assumed that all the As speciation in the present study were the most toxic inorganic forms. For As, approximately 0.54% of consumers who consumed *Pheretima* were at non-carcinogenic risk (Figure 2B).

#### 3.3.2 Carcinogenic Risk Assessment of Heavy Metals in *Pheretima*

**FIGURE 3:**
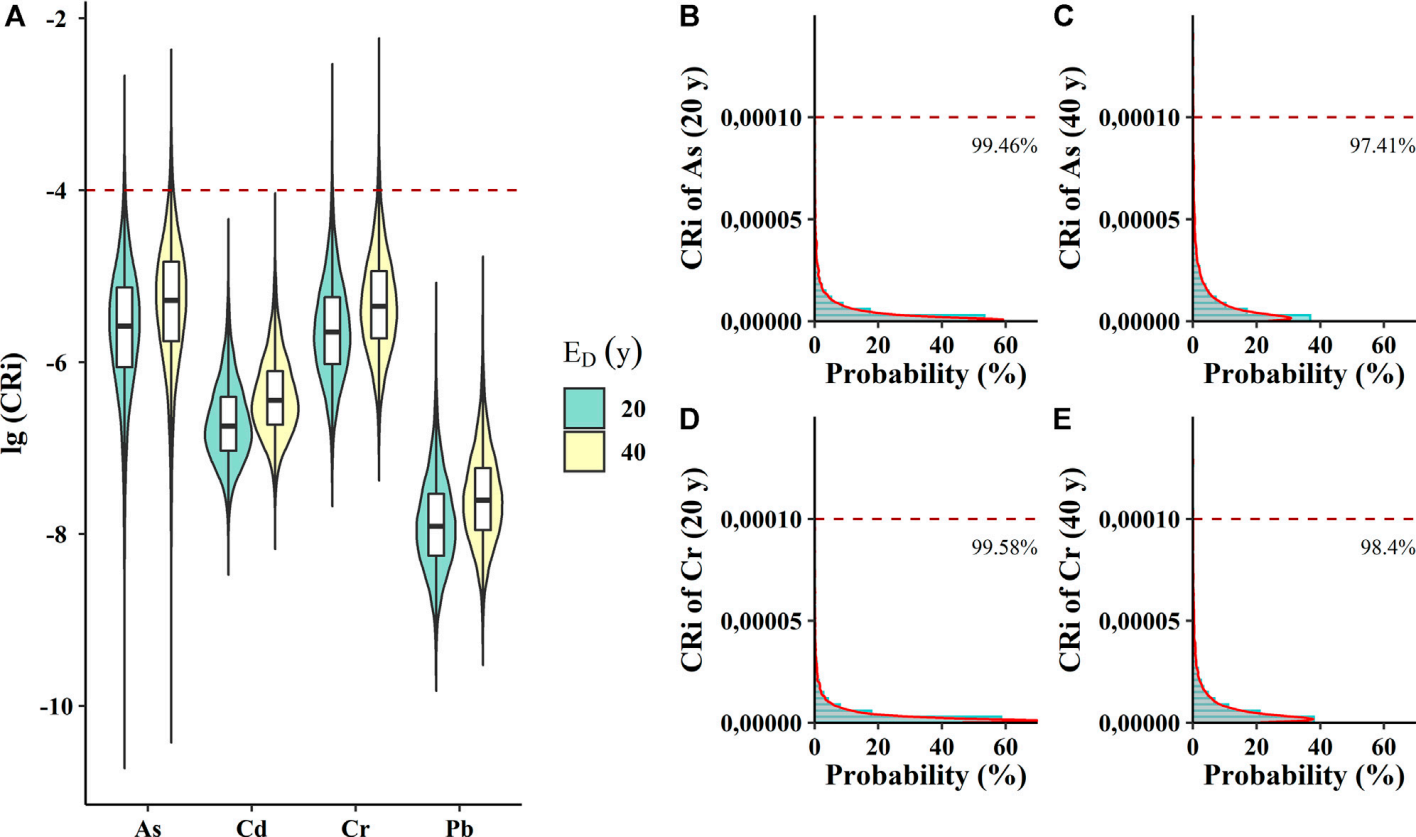
Estimated carcinogenic risks (CR_i_) of selected heavy metals in *Pheretima*. **(A)** Violin plot showing log_10_ (CR_i_) values of eight heavy metals in *Pheretima*. **(B,C)** The probability distribution of potential health risk of As during the exposure of 20 and 40 years. **(D,E)** The probability distribution of potential health risk of Cr during the exposure of 20 and 40 years. The red dashed line the acceptable limit of CR_i._.

With the exposure duration assumed to be 20 and 40 years, Figure 3 presents the results of carcinogenic risk levels of four heavy metals in *Pheretima* based on probabilistic health risk assessments. Apparently, carcinogenic risk levels for 40 years of exposure duration were greater than those for 20 years of exposure duration. The median levels for carcinogenic risk in *Pheretima* were observed in the descending order of As > Cr > Cd > Pb. As and Cr were regarded as the primary contributors to carcinogenic risks in *Pheretima*. When the exposure duration was 20 years, 0.54 and 0.43% of *CRi* values were higher than the carcinogenic risk of As and Cr, respectively (Figures 3B,D). If a patient consumes Pheretima for more than 40 years, 2.64 and 1.60% of CRi values will be higher than the acceptable risk of As and Cr, respectively (Figures 3C,E). Therefore, special attention should be taken to prioritize reductions in these heavy metals, especially for As and Cr in *Pheretima*, as they may be adversely affecting the health of some users.

#### 3.3.3 Comparison of the Comprehensive Results of Probabilistic and Deterministic Health Risk Assessments

To compare the difference between probabilistic and deterministic health risk assessments, further analysis of non-carcinogenic risks and carcinogenic risks for *Pheretima* was carried out, as given in Supplementary Tables S4, S5. It was apparent from these tables that the outcome of the probabilistic assessments was an interval, rather than a single value for that of a deterministic assessment.

**FIGURE 4:**
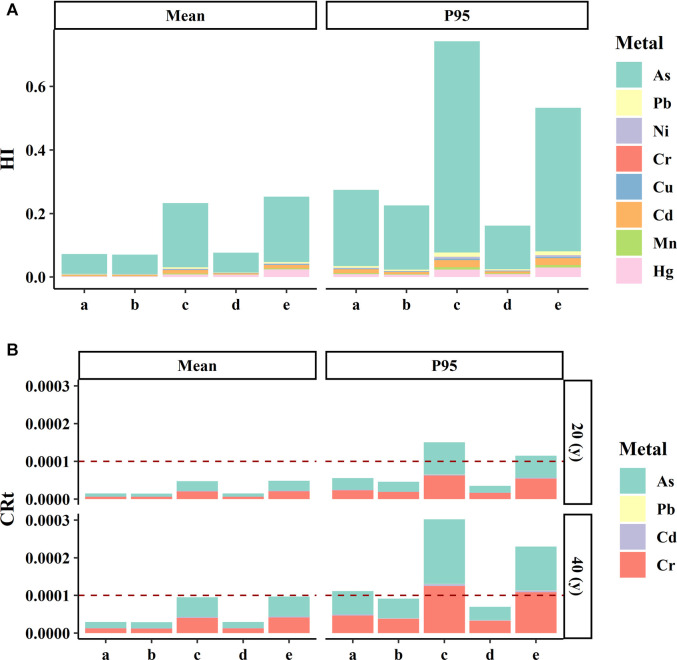
The comparison of the comprehensive results of probabilistic and deterministic health risk assessment. **(A)** Comparison of the estimated non-carcinogenic risks (HQ) results of probabilistic and deterministic health risk assessment. **(B)** Comparison of the estimated carcinogenic risks (CR_i_) results of probabilistic and deterministic health risk assessment. The letters a, b, c, d, e represent the probabilistic health risk assessment for random users, the probabilistic health risk assessment for average users, the probabilistic health risk assessment for high users, the deterministic health risk assessment for average users, the deterministic health risk assessment for high users, respectively. Red dashed lines: the acceptable limit of HQ and CR_i_, respectively.

The results, as shown in Figure 4A, indicate that non-carcinogenic risks based on probabilistic assessment for random users are broadly consistent with those of average users. The mean value of probabilistic assessment of HI for frequent users is lower than that of deterministic assessment, but the P95 value of probabilistic assessment of HI is significantly higher than that of the deterministic assessment. Both HQ and HI were less than 1, which indicates no obvious non-carcinogenic risks from these heavy metals. In both probabilistic and deterministic health risk assessments, As was regarded as the primary contributor to non-carcinogenic risks in *Pheretima*.

When it came to carcinogenic risks (Figure 4B), there were some differences between the two assessment methods. When the exposure duration was 20 years, both the mean and P95 of the carcinogenic risk for random and average consumers were within the acceptable level according to the USEPA, whereas the 95th percentile of *CR*_*T*_ exposed to four carcinogenic contaminants (As, Cd, Cr, and Pb) for frequent users exceeded the acceptable level. The results suggest that approximately over 5% of integrated carcinogenic risks posed by *Pheretima* for frequent users are beyond the acceptable level (1 × 10^−4^). When the exposure duration was extended to 40 years, the mean of the carcinogenic risk of all consumers was still lower than the acceptable level. However, the P95 of *CR*_*T*_ for random consumers based on the probabilistic risk assessments was slightly higher than the acceptable level; the P95 of *CR*_*T*_ for frequent users based on the probabilistic and deterministic risk assessments was significantly higher than acceptable levels. In both probabilistic and deterministic health risk assessments, As and Cr were regarded as the primary contributors to carcinogenic risk in *Pheretima*. These findings draw our attention to the importance of considering health risks caused by *Pheretima*.

### 3.4 Estimation of the Exposure Frequency of Safety

**FIGURE 5:**
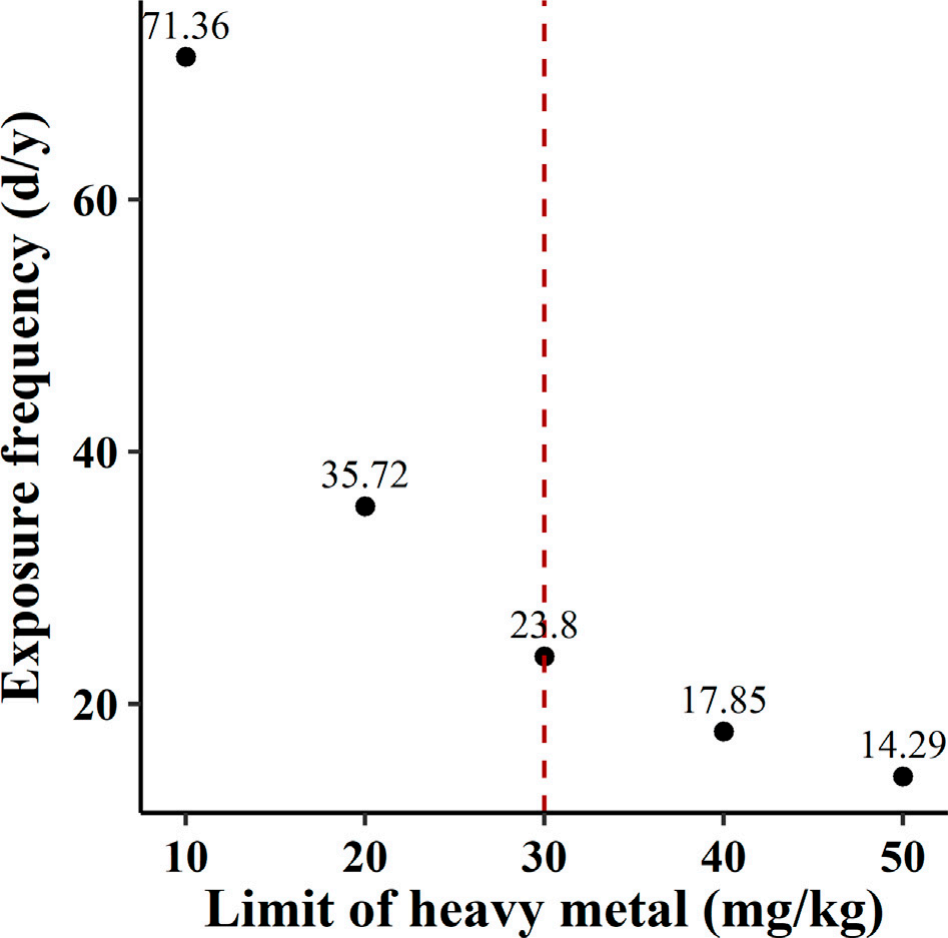
Exposure frequency for safety under different metal limit standards. The dashed line represents the limits set in the Chinese Pharmacopoeia 2020 (ChP).

Based on the aforementioned results, As has the highest risk value as found from the carcinogenic and non-carcinogenic risk assessments. Therefore, As was chosen to set the safe exposure duration. The expressions used to calculate the frequency of exposure are as follows:

$$\begin{array}{c} EDI_{max}=\frac{Risk_{l}\times SF}{CSF_{As}} \end{array}\;$$ (6)

$$\begin{array}{c} EF=\frac{EDI_{max}\times T_{A}\times BW\times 1000}{SF\times C_{l}\times E_{D}\times T\times RDI} \end{array},$$ (7)

where *EDI*_*max*_ is the maximum estimated daily intake of heavy metals under the carcinogenic risk limit, *Risk*_*l*_ is the acceptable carcinogenic risk criteria (1.00 × 10^−4^), and *SF* represents safe factor, which is the ratio of TCM to total food. It is suggested that the value of *SF* is 13.3% (Zuo et al., 2019). According to the Chinese Pharmacopoeia 2020 (ChP), the maximum recommended daily intake (*RDI*) of *Pheretima* is 10 g/d, and the maximum metal concentration limit (*C*_*l*_) in *Pheretima* is 30 mg/kg. The expression for carcinogenicity and the maximum recommended intake of *Pheretima* were used to calculate the exposure period to As, under the corresponding limits. The distribution of metal limit and exposure frequency are shown in Figure 5, which shows that the dosing frequency should be less than 24 d per year when the limit is set at 30 mg/kg.

## 4 Discussion

This study is designed to investigate the contents of heavy metals in 98 batches of *Pheretima* from Chinese pharmaceutical markets and assess related probabilistic health risks. To date, questionnaire assessments of TCM have been traditionally employed in the past to investigate the consumption of TCM. For example, Zuo et al. (2020) assessed the risk of many herbal medicines using the results of all the TCMs using a questionnaire. In fact, it is rather difficult to investigate the consumption data of all types of TCMs through questionnaires owing to a large variety of TCMs in the current market, and most consumers do not pay attention to the ingredients of TCMs they take. Besides, these results of questionnaire assessments are clearly limited by the relatively subjective samples. Here, we analyzed the supply chain data of *Pheretima* from Shanghai pharmaceutical markets and hospitals, which could provide a more objective result for further investigation.

Recently, there has been an increasing interest in heavy metal concentration in TCM (Liu et al., 2015). Yang et al. (2021) calculated the non-carcinogenic toxicity in 279 types of Chinese herbal medicines (CHMs) by using the deterministic method, which showed that *Laminaria thallus*, *Spirodela herba*, and *Indigo naturalis* possessed a high degree of risk of As contamination. Zuo et al. (2020) developed a deterministic risk assessment strategy to obtain the non-carcinogenic and carcinogenic toxicity in 32 types of CHMs, which showed that As and Hg contributed greatly to the non-carcinogenic risk and As is associated with argy wormwood leaf, morinda root, plantain herb, Chinese angelica, common coltsfoot flower, long-tube ground ivy herb, Indian madder root, sencha, dyers woad leaf, and perilla leaf should be paid more attention for a carcinogenic risk. This study found that the risk of As and Cr should be concerned in *Pheretima*, which is consistent with that of Zuo et al. (2019), who found that As was regarded as potentially hazardous in the context of continuous *Pheretima* use by the method of deterministic health risk assessments. As is a ubiquitous metalloid, which could induce toxicity at low level of exposure (Duffus, 2002). The adverse health effects associated with inorganic As include cardiovascular problems, kidney dysfunction, muscle spasms, peripheral neuritis, hematologic disorders, immune system diseases, and increased risk of stillbirth in pregnant women (Chung et al., 2013; Yang et al., 2013). Furthermore, epidemiological studies have indicated As exposure is linked to increased risk of cancer in the liver, kidney bladder, and lungs (Chen et al., 1992). Cr is widely used in numerous industrial processes, such as industrial welding, chrome plating, dyes and pigments, leather tanning, and wood preservation (Cohen et al., 1993). Studies have indicated that Cr (VI) is cytotoxic and able to induce DNA-damaging effects such as chromosomal abnormalities (Patlolla et al., 2008), DNA strand breaks, DNA fragmentation, and oxidative stress in Sprague–Dawley rats and human liver carcinoma cells (Patlolla et al., 2009; Kumar et al., 2013). Earthworms can bioaccumulate high concentrations of metals, including heavy metals, in their tissues without affecting their physiology, so they could particularly ingest and enrich extremely high amounts of heavy metals (Ghomi Avili et al., 2018). As a consequence, the aforementioned reasons may cause excessive metal concentration in *Pheretima* and then pose health risk to the exposed population.

When deterministic parameters are used in risk assessment, uncertainty may occur. Therefore, this study conducted a probabilistic health risk assessment on TCMs. A Monte Carlo simulation was applied to obtain a range of predicted values and then reduce the uncertainty during the process of risk assessments (Mari et al., 2009; Tong et al., 2018). The corresponding probabilistic risk assessments were carried out with the 10th, 25th, 50th, 75th, 90th, 95th, and 97.5th percentile values as well as the minimum and maximum values. These results mirror those of the previous studies that the results with Monte Carlo simulations can contain more information than a single value used in a deterministic method before.

Considering the public confidence in the safe use of traditional animal medicines, the development of a scientific and practical heavy metal standard for traditional animal medicines is encouraged. Heavy metal safety limit guidance values in *Pheretima* and recommended maximum daily intake of *Pheretima* has been recorded in the Chinese Pharmacopoeia 2020 (ChP). Based on the aforementioned guidelines, the present study has provided additional evidence with respect to the recommended frequency of intake within a year for consuming *Pheretima*, which proves to be also particularly valuable to the doctors of TCM.

Despite these promising results, the questions remain. The findings are subject to at least three limitations. Firstly, the absorption rate of heavy metals was assumed to be 100% in the present study. These results are from the safest perspective for users, therefore, may overestimate health risks exposed by *Pheretima*. As is mentioned in the literature review; the oral bioavailability of metals in complementary medicines depends on speciation of these metals (Bolan et al., 2017). Recent study suggests low relative bioavailability (RBA) of As and Hg after taking the *Liu Shen Wan*, which is a well-known Chinese formula used in treating infectious diseases (Tinggi et al., 2016). However, the research vacancy of RBA in Chinese animal medicine still remains to be explored, especially for the priority metal Cr and As. Secondly, this investigation has only considered the consumption data and the metal concentrations of *Pheretima*, when performing the Monte Carlo simulation. However, specific parameter selections (E_D_, BW, T_A_, and T) depended on existing findings. It is not accurate enough to represent the whole receptors with a single value of a specific parameter. Besides, factors such as gender and age were not considered in this study. Further research regarding the role of these parameters would be a useful way to better understand the safety of TCM. The communication between relevant research institutions and hospitals will need to be strengthened to obtain the age and gender of anonymous TCM consumers for improving the accuracy of risk assessment. Thirdly, the risk assessment did not consider the cumulative exposure to other environmental pollutants with a toxicity mechanism similar to that of the heavy metals. The co-existence of such pollutants will affect the risk assessment of daily metal exposure. Therefore, systematic studies with larger scale sampling are necessary to expand the database of heavy residues in TCMs. Therefore, larger scale data of specific TCM consumption and corresponding heavy metal concentration are necessary to expand the risk assessment database of heavy metals in TCMs. Taken together, the study certainly makes several noteworthy contributions that have a bearing on probabilistic health risks that arises by exposure to *Pheretima* and provides a basis for a deeper insight into this field.

In general, this appears to be the first study to evaluate the health risks of traditional animal medicines from Chinese markets with a probabilistic assessment. These findings are particularly relevant to both policy makers and consumers. On the one hand, policy makers can place more emphasis on As and Cr since these are the primary metals to carcinogenic risk in the current study, when considering the safety of exogenous ingredients in *Pheretima*. On the other hand, some measures should be taken by consumers to control the health risks. For instance, people can extend the frequency of intake for some TCMs with higher risk. Taken together, it is necessary to pay more attention to this topic and take some effective measures to prevent these heavy metals in *Pheretima* from causing greater health damage.

## 5 Conclusion

In the present study, heavy metals were analyzed in 98 batches of *Pheretima* samples collected from Chinese markets, and the probabilistic health risks of heavy metals in *Pheretima* were evaluated based on the consumption data of Shanghai. The findings clearly found that the mean concentrations of heavy metals in *Pheretima* were observed in the descending order of Mn > Cr > Cu > As > Pb > Ni > Cd > Hg. The probabilistic assessment of dietary exposure showed that the non-carcinogenic risk of As is higher than the acceptable level, and the carcinogenic risk levels of As and Cr exceeded the 1 × 10^−4^ threshold. It was recommended that the dosing frequency should be less than 24 d/y. In addition, considering the widespread presence of heavy metals, other TCMs should also be evaluated with the heavy metal–related health risks using similar methods. It is also necessary to consistently study the coexistence of exposure pathways of heavy metals in patients, specify the limits for heavy metals in TCMs, and standardize TCM agriculture to monitor its risk to human beings.

## Data Availability

The original contributions presented in the study are included in the article/Supplementary Material, further inquiries can be directed to the corresponding authors.

## Abbreviations

CSF, cancer slope factor; ED, exposure duration; EDI, estimated daily intake; EF, exposure frequency; HQ, hazard quotient; LOD, limit of detection; LOQ, limit of quantitation; RDI, recommended daily intake; RfD, reference dose; RSD, relative standard deviation; TCM, traditional Chinese medicine.
