# Supplementary material for: Heavy Metals and Probabilistic Risk Assessment via Pheretima (a Traditional Chinese Medicine) Consumption in China
Source: Front Pharmacol. 2022 Jan 5;12:803592. doi: 10.3389/fphar.2021.803592 (PMC8767006; doi:10.3389/fphar.2021.803592)
Supplement: Supplementary file 1 [file DataSheet1.docx]

Supplementary Material for Heavy metals and probabilistic risk assessment via Pheretima (a Traditional Chinese Medicine) consumption in China

# Supplementary Tables

**Table S1.** Operating parameters of Agilent 7700 ICP -MS

| Parameter | Value |
| --- | --- |
| RF Power | 1500 W |
| Sample Depth | 8.0 mm |
| Plasma Ar flow rate | 15.0 L·min^-1^ |
| Auxiliary Ar flow rate | 1.0 L·min^-1^ |
| Nebulizer Gas | 0.8 L·min^-1^ |
| Makeup Gas | 0.4 L·min^-1^ |
| Nebulizer Pump | 0.10 mL·min^-1^ |
| S/C Temp | 2℃ |
| Acq Mode | Spectrum |
| Peak Pattern | 3 |
| Number of replicate | 3 |
| Integration time | 1.0 s for As, Cd, 3.0 s for Hg, 0.3 s for other elements |
| Reaction cell | 4.3 mL·min^-1^ |

**Table S2.** The LOD, LOQ, repeatability and recovery of the method

| Elements | Limits of Detection （mg/kg） | Limits of Quantification （mg/kg） | repeatability RSD (%) | Recovery | Recovery RSD (%) |
| --- | --- | --- | --- | --- | --- |
| ^53^ Cr | 0.135 | 0.450 | 4.3 | 100.9% | 10.8 |
| ^55^ Mn | 0.026 | 0.087 | 3.8 | 108.1% | 8.1 |
| ^60^ Ni | 0.033 | 0.110 | 4.7 | 95.5% | 10.0 |
| ^63^ Cu | 0.022 | 0.073 | 4.5 | 95.3% | 8.1 |
| ^75^ As | 0.015 | 0.050 | 5.5 | 87.5% | 13.8 |
| ^111^ Cd | 0.002 | 0.007 | 17.8 | 80.1% | 1.2 |
| ^202^ Hg | 0.014 | 0.047 | 19.1 | 113.0% | 2.2 |
| ^208^ Pb | 0.021 | 0.070 | 8.8 | 99.6% | 5.1 |

**Table S3.** Heavy metal pollution in *Pheretima* (mg/kg)

| Metal | Mean | SD^a^ | Min | Max | Median | P95 | ER^b^ | Distribution type |
| --- | --- | --- | --- | --- | --- | --- | --- | --- |
| As | 13.51 | 20.19 | 0.19 | 130.64 | 10.61 | 29.69 | 5.10% | weibull(shape=0.88, scale=12.57) |
| Cd | 2.52 | 1.84 | 0.34 | 16.93 | 2.28 | 4.81 | 0% | lnorm(meanlog=0.78, sdlog=0.51) |
| Cr | 31.85 | 46.46 | 2.29 | 423.18 | 21.41 | 82.98 | 35.71% | lnorm(meanlog=3.00, sdlog=0.95) |
| Cu | 17.23 | 20.27 | 3.83 | 140.49 | 12.90 | 32.97 | 6.12% | lnorm(meanlog=2.58, sdlog=0.64) |
| Hg | 1.58 | 11.07 | 0.10 | 109.87 | 0.31 | 1.99 | 1.02% | lnorm(meanlog=-1.03, sdlog=0.89) |
| Mn | 83.45 | 60.49 | 18.59 | 274.16 | 65.30 | 220.32 | 82.65% | invGauss(nu=83.44, lambda=137.37) |
| Ni | 8.05 | 8.56 | 0.75 | 45.20 | 4.57 | 22.34 | 3.06% | lnorm(meanlog=1.65, sdlog=0.93) |
| Pb | 9.33 | 10.19 | 1.46 | 76.51 | 6.42 | 23.08 | 3.06% | lnorm(meanlog=1.88,sdlog=0.81) |

^a^ SD represents standard deviation.

^b^ ER represents the exceedance ratios.

**Table S4.** Comparison of non-carcinogenic risks of trace elements in *Pheretima* based on probabilistic and deterministic assessments.

| Note | Assessment_Methods | User | Value | As | Pb | Ni | Cr | Cu | Cd | Mn | Hg | HI |
| --- | --- | --- | --- | --- | --- | --- | --- | --- | --- | --- | --- | --- |
| A | Probabilistic assessment | Random | Mean | 6.43e-02 | 1.55e-03 | 5.52e-04 | 2.82e-05 | 5.54e-04 | 3.48e-03 | 8.19e-04 | 2.37e-03 | 7.37e-02 |
|  |  |  | P95 | 2.44e-01 | 6.03e-03 | 2.06e-03 | 1.03e-04 | 1.96e-03 | 1.22e-02 | 2.88e-03 | 8.90e-03 | 2.78e-01 |
| B |  | Average users | Mean | 6.24e-02 | 1.50e-03 | 5.52e-04 | 2.89e-05 | 5.55e-04 | 3.46e-03 | 8.15e-04 | 2.45e-03 | 7.18e-02 |
|  |  |  | P95 | 2.07e-01 | 4.13e-03 | 1.64e-03 | 8.68e-05 | 1.28e-03 | 6.94e-03 | 2.06e-03 | 7.42e-03 | 2.31e-01 |
| C |  | High users | Mean | 2.06e-01 | 4.94e-03 | 1.82e-03 | 9.51e-05 | 1.83e-03 | 1.14e-02 | 2.69e-03 | 8.06e-03 | 2.36e-01 |
|  |  |  | P95 | 6.82e-01 | 1.36e-02 | 5.40e-03 | 2.86e-04 | 4.23e-03 | 2.28e-02 | 6.78e-03 | 2.44e-02 | 7.59e-01 |
| D | Deterministic assessment | Average users | Mean | 6.24e-02 | 1.52e-03 | 5.58e-04 | 2.94e-05 | 5.97e-04 | 3.49e-03 | 8.27e-04 | 7.30e-03 | 7.68e-02 |
|  |  |  | P95 | 1.37e-01 | 3.76e-03 | 1.55e-03 | 7.67e-05 | 1.14e-03 | 6.67e-03 | 2.18e-03 | 9.18e-03 | 1.62e-01 |
| E |  | High users | Mean | 2.06e-01 | 5.01e-03 | 1.84e-03 | 9.70e-05 | 1.97e-03 | 1.15e-02 | 2.72e-03 | 2.40e-02 | 2.53e-01 |
|  |  |  | P95 | 4.52e-01 | 1.24e-02 | 5.10e-03 | 2.53e-04 | 3.76e-03 | 2.20e-02 | 7.19e-03 | 3.02e-02 | 5.33e-01 |

**Table S5.** Comparison of carcinogenic risks of trace elements in *Pheretima* based on probabilistic and deterministic assessments.

| Note | Assessment_Methods | User | ED | Value | As | Cd | Cr | Pb | CR_T_ |
| --- | --- | --- | --- | --- | --- | --- | --- | --- | --- |
| A | Probabilistic assessment | Random | 20 | Mean | 8.27e-06 | 3.83e-07 | 6.39e-06 | 3.20e-08 | 1.51e-05 |
|  |  |  |  | P95 | 3.14e-05 | 1.34e-06 | 2.36e-05 | 1.17e-07 | 5.64e-05 |
|  |  |  | 40 | Mean | 1.65e-05 | 7.65e-07 | 1.28e-05 | 6.40e-08 | 3.02e-05 |
|  |  |  |  | P95 | 6.28e-05 | 2.68e-06 | 4.72e-05 | 2.33e-07 | 1.13e-04 |
| B |  | Average users | 20 | Mean | 8.03e-06 | 3.75e-07 | 6.19e-06 | 3.10e-08 | 1.46e-05 |
|  |  |  |  | P95 | 2.66e-05 | 7.53e-07 | 1.86e-05 | 8.53e-08 | 4.61e-05 |
|  |  |  | 40 | Mean | 1.61e-05 | 7.50e-07 | 1.24e-05 | 6.20e-08 | 2.92e-05 |
|  |  |  |  | P95 | 5.32e-05 | 1.51e-06 | 3.72e-05 | 1.71e-07 | 9.21e-05 |
| C |  | High users | 20 | Mean | 2.64e-05 | 1.24e-06 | 2.04e-05 | 1.02e-07 | 4.81e-05 |
|  |  |  |  | P95 | 8.76e-05 | 2.48e-06 | 6.12e-05 | 2.81e-07 | 1.52e-04 |
|  |  |  | 40 | Mean | 5.29e-05 | 2.47e-06 | 4.08e-05 | 2.04e-07 | 9.63e-05 |
|  |  |  |  | P95 | 1.75e-04 | 4.96e-06 | 1.22e-04 | 5.62e-07 | 3.03e-04 |
| D | Deterministic assessment | Average users | 20 | Mean | 8.03e-05 | 3.79e-06 | 6.31e-05 | 3.14e-07 | 1.47e-04 |
|  |  |  |  | P95 | 1.76e-04 | 7.24e-06 | 1.64e-04 | 7.77e-07 | 3.49e-04 |
|  |  |  | 40 | Mean | 1.61e-04 | 7.59e-06 | 1.26e-04 | 6.28e-07 | 2.95e-04 |
|  |  |  |  | P95 | 3.53e-04 | 1.45e-05 | 3.29e-04 | 1.55e-06 | 6.98e-04 |
| E |  | High users | 20 | Mean | 2.64e-04 | 1.25e-05 | 2.08e-04 | 1.03e-06 | 4.86e-04 |
|  |  |  |  | P95 | 5.81e-04 | 2.39e-05 | 5.41e-04 | 2.56e-06 | 1.15e-03 |
|  |  |  | 40 | Mean | 5.29e-04 | 2.50e-05 | 4.16e-04 | 2.07e-06 | 9.71e-04 |
|  |  |  |  | P95 | 1.16e-03 | 4.77e-05 | 1.08e-03 | 5.12e-06 | 2.30e-03 |

# Supplementary Figures

**
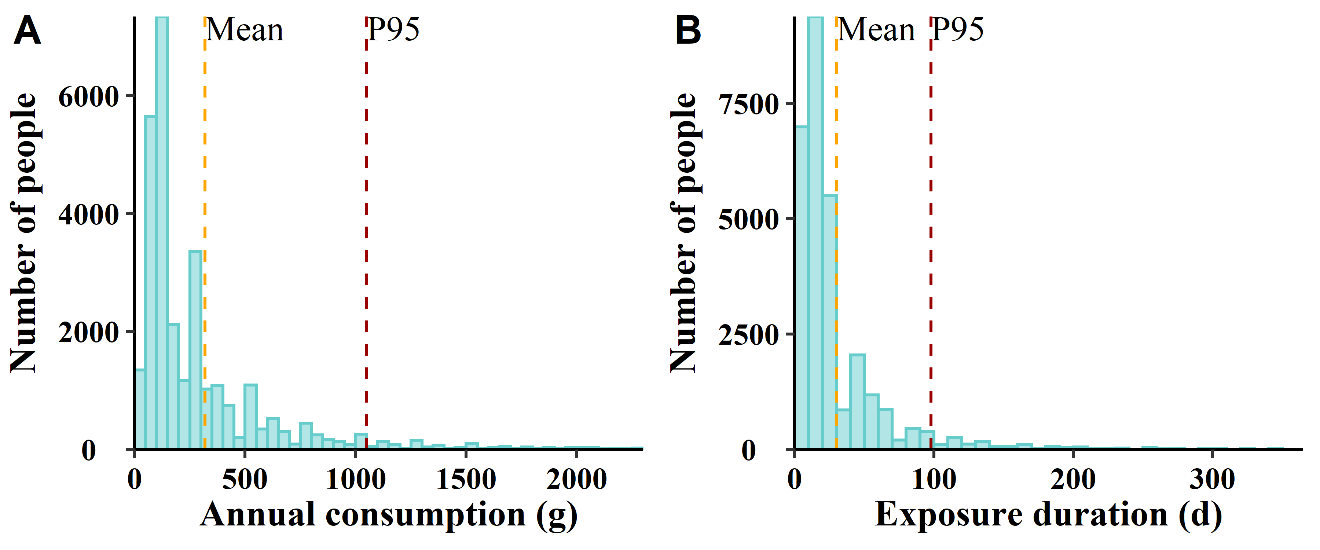
**

**Supplementary Figure 1.** The distribution of annual consumption and exposure frequency
